# Quantifying uncertainty in time perception: A modified reproduction method

**DOI:** 10.1177/20416695241279675

**Published:** 2024-12-08

**Authors:** Jaume Boned, Joan López-Moliner

**Affiliations:** Institut de Neurociències, 16724 Universitat de Barcelona, Barcelona, Spain

**Keywords:** time perception, reproduction, uncertainty, quantitative timing, time estimation

## Abstract

In time perception research, we typically measure how an observer perceives time intervals by collecting data from multiple trials with a single estimate recorded on each. However, this gives us limited information about the observer's uncertainty for each estimate, which we usually measure from the variability across trials. Our study tested the potential of a modified reproduction task to provide a duration estimate as well as a measure of uncertainty on a single-trial basis. Participants were instructed to press and hold a key to temporally bracket the end of a learned duration (0.6–4 s) as narrowly as possible. Therefore, we expected the bracket's length to indicate the level of uncertainty. We compared this method to a conventional reproduction task. Taking the mid-point of the bracket as the duration estimate, we found that both methods produced equivalent data. Critically, the bracket length predicted reproduction variability, indicating that a single bracket obtained in an individual trial could potentially provide as much information as multiple reproductions. Additionally, relative variability in bracket start and end positions suggests a combination of additive and multiplicative noise components. Our findings highlight the bracket method as a more efficient and nuanced approach to measure time estimates and their associated uncertainty, expanding the methodological toolkit and opening new avenues in time perception research.

The study of time perception plays a critical role in understanding human cognition and behavior. It aims to shed light on how individuals measure, interpret, and interact with the temporal aspects of their environment, making it a pivotal aspect of our daily life. For this reason, using adequate methods for measuring time perception is essential to this field.

Over the years, various methods have been used, broadly categorized into prospective and retrospective methods. While prospective tasks require participants to actively think about time, involving attentional and executive control components, retrospective tasks rely mainly on memory, as participants are unaware that they will be asked about time until after the event (Block & Zakay, 1997; Vatakis et al., 2018). Another key distinction lies in tasks that yield quantitative measures versus those based on perceptual judgments. A commonality between all these methods is that measures of time perception can always be prone to sources of noise. Reproduction tasks, a form of quantitative measures, may depend on motor processes; judgment tasks are prone to decisional biases, which might be challenging to distinguish from perceptual biases (Ueda et al., 2022; Wehrman et al., 2020); and estimation or production tasks are also susceptible to anchoring or rounding effects (Damsma et al., 2021; Tobin et al., 2010; Vatakis et al., 2018).

This highlights that all methods can be subject to different sources of interference that can introduce variability in participants’ measures of time perception. This noise can arise from both internal and external factors, and it manifests uncertainty in responses. Here, uncertainty refers to the degree of variability or unreliability in participants’ time estimates and being able to obtain a measure of it is key to understanding the complexity of time perception. To address this, it is necessary to develop methods that can accurately quantify uncertainty, enabling researchers to understand and mitigate the factors contributing to the noisy and often inconsistent estimates observed in time perception studies.

Probably, the most common approach is to infer the uncertainty from the variability in responses using quantitative tasks. For example, in the reproduction task, participants are asked to estimate a specific time interval behaviorally, often by performing an action for as long as the interval should last or by explicitly pinpointing the offset, and in some cases, also the onset (Vatakis et al., 2018). For example, this could involve pressing a key for a given time interval or, for instance, tapping twice to mark the beginning and end of an interval lasting that amount of time. Here, uncertainty is expected to be expressed as an increased variability in the reproduction estimates. However, it is challenging to discern how much of this variability actually reflects uncertainty as opposed to other factors. Additionally, it requires as much data as possible to ensure a robust measure of variability.

A similar, but more elaborate, approach is the one used with the peak-interval (PI) procedure. In this type of task, participants (usually non-human animals) are trained on a fixed-interval schedule of reinforcement where a response (i.e., a lever press) is rewarded after a set period. This method reveals a distribution of response rates peaking around the reinforcement time. The spread of response rates serves as an informative measure of uncertainty regarding the proximity of the current time to the reinforced interval. Regarding this response variability, studies in alignment with the scalar expectancy theory (SET) highlight how the scalar property of timing is manifested in this case, where response variability is proportional to the target interval, suggesting a direct relation between the spread of response rates and uncertainty (Gibbon & Church, 1990; Rakitin et al., 1998). However, despite the fact that this measure of uncertainty can be obtained in each individual trial, the PI procedure's reliance on extensive training and a reward system poses challenges for its application across species and paradigms, despite evidence of its generalizability (Rakitin et al., 1998). The necessity for multiple responses per trial and the complexity of adapting this system to different experimental setups underscore its limitations.

Another method that is conceptually similar to the peak-interval procedure, but keeps the simplicity of the reproduction task, is the start–stop procedure. Introduced by Kladopoulos et al. (1998), it is a method for estimating sample durations that, rather than delivering a discrete reproduction time, provides a bracketed interval consisting of two timepoints around the target time. In contrast to the traditional reproduction task, here, participants were asked to “terminate the estimation stimulus by pressing the response button when they estimated that the sample duration was about to elapse and release it when they estimated that the sample duration had elapsed.” The two-point response should always bracket the termination time while aiming to “create the smallest possible bracket without missing the sample duration.”

These two latencies represent the start and stop times and the interval between these two points in time provides a “bracket” that allows the estimation on each individual trial of the point of subjective equality (PSE) and difference limen (DL) determined by the allocation in time and length of the bracket, respectively. The difference limen in this context is analogous to the standard deviation of the temporal estimates obtained after multiple trials in a classic reproduction task, thus serving as a potential measure of uncertainty. In summary, this procedure differs from the classic reproduction as it yields new measures, start and stop times on individual trials instead of only one reproduction estimate, and from the peak-interval procedure as it provides direct measures of these variables and does not have to be inferred from response distributions. Balcı et al. (2013) followed a similar method to assess how dopamine variations impact the relationship between reward processing and perception of time intervals. In their study, participants were asked to initiate their responses in anticipation of a reward and maintain them until the expected time of reward delivery, as with the start–stop procedure. They found that larger reward magnitudes were related to earlier initiation of responses, producing an increase in both the width of these responses and the estimates variability. This suggests that the width of the brackets and the coefficient of variation, as measures of timing uncertainty, are influenced by reward magnitude and points out the width of the brackets as a good candidate for representing uncertainty in timing tasks.

Grounded in this principle that subjective time can encompass a range of values rather than a singular estimate, other studies have adopted a verbal estimation technique that also captures subjective durations within a range. Grondin and colleagues (Bisson & Grondin, 2013; Grondin & Plourde, 2007; Tobin et al., 2010; Tobin & Grondin, 2012) explored this by asking participants to give maximum and minimum duration estimates considered equal to already experienced durations. By using this approach in retrospective tasks with especially longer durations, they demonstrated its utility to measure variability, and therefore uncertainty, in tasks where traditional metrics might fall short due to the smaller number of observations.

Even studies in other modalities also designed tasks where responses were estimations delivered as a range instead of a discrete value and used this as a measure of uncertainty. For example, Graf et al. (2005) assessed whether humans could estimate their own uncertainty in extrapolating motion trajectories of objects that followed a random walk. They asked participants to delimit a “capture region” where they predicted an object would reappear after being momentarily occluded and adjust the width of this area according to their uncertainty. This adjustment was based on the varying directional variability of the object's movement, with participants effectively compensating for different levels of unpredictability in the object's path. Similarly, in another study by Honig et al. (2020), participants were asked to select a color that they were presented previously by drawing an arc on a color wheel. Here, the width of the arc served as a metric for uncertainty, indicating participant's confidence in their memory of the color.

Following this rationale, we conceive the start–stop procedure as a method that acknowledges the perception of time not as a point estimate, but as an interval, offering a more realistic representation of how time is perceived and processed and particularly useful in understanding the variability in duration estimates and the underlying cognitive processes involved in timing behavior. However, this method has not been directly assessed as a tool for measuring uncertainty or compared directly with the classic discrete reproduction method. Because of this, the aim of our study is to compare the start–stop procedure with the classic discrete reproduction method to evaluate their equivalency in measuring time perception as well as to assess the start–stop procedure's potential as a direct measure of uncertainty in time estimates grounded in theoretical framework. This comparison will provide deeper insights into the accuracy and utility of these methods in understanding the underlying processes of time perception and its associated uncertainties.

## Method

### Participants

Fourteen individuals from the University of Barcelona took part in the study (six females and eight males, *M*_age_ = 28.36, SD = 4.38). All participants provided written informed consent and were initially naïve to the purpose of the experiment and had normal or corrected-to-normal vision. The study is part of a research program approved by the ethical committee of the University of Barcelona (IRB00003099), according to the principles stated in the Declaration of Helsinki.

### Apparatus and Stimuli

**Figure 1.**
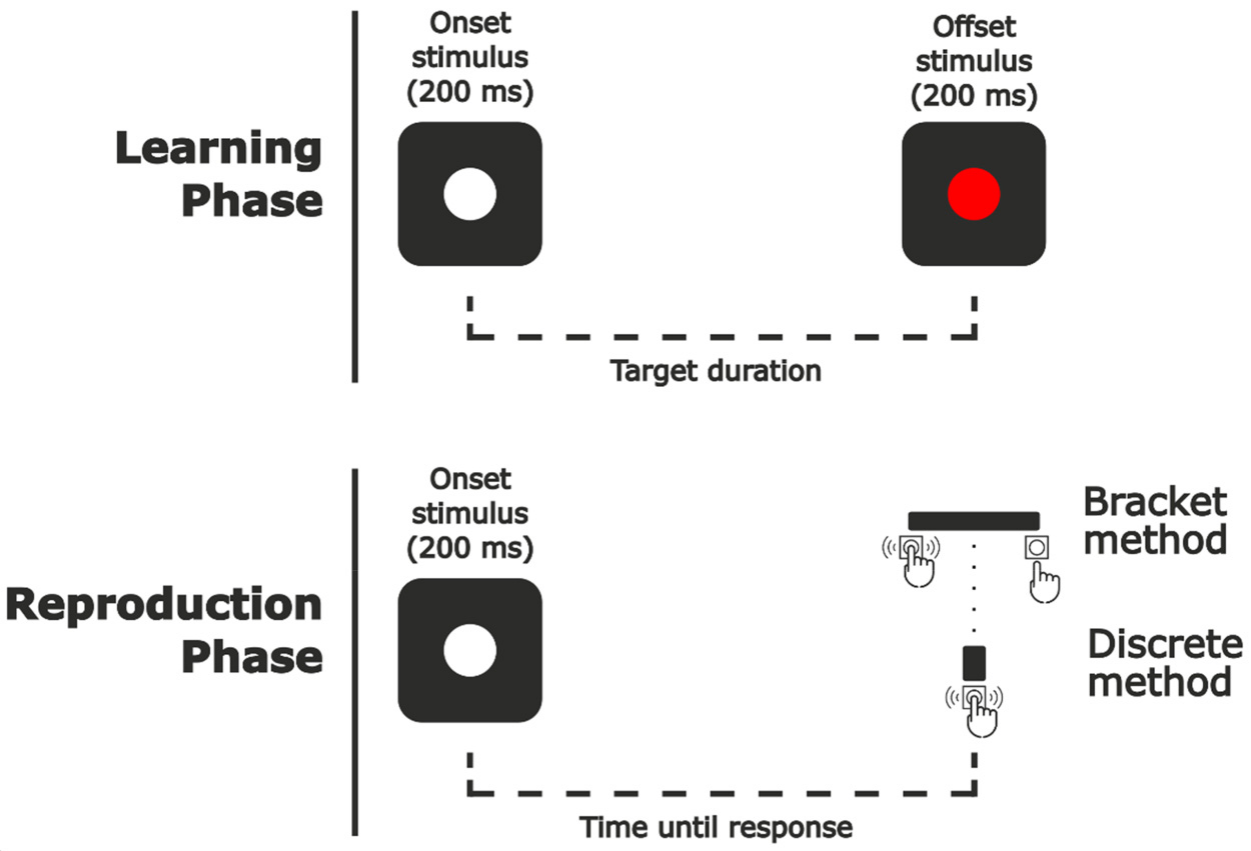
Sequence of the reproduction task. Reproduction phase ended at the onset of the key press during the discrete reproduction blocks and at the offset of the key press during the bracket reproduction blocks.

The task was designed using Unity 2020.3.27f1. Stimuli were presented on a 24.5 in. ASUS ROG Swift PG258Q monitor with a resolution of 1920 × 1080 px at a refresh rate of 240 Hz. Participants maintained a distance of 57 cm from the screen. Visual stimuli consisted of white and red disks with a diameter of 3° against a gray background (see Figure 1).

### Procedure

Each trial was divided into two phases: a learning phase and a reproduction phase.

In the learning phase, a white cross was presented in the center of the screen (2°) and remained until participants’ response of the CTRL key, at which point it disappeared. After 1500 ms delay, a white disk (3° diameter) was presented for 200 ms at a random location within a 12 × 12° area centered on the screen. After an interval determined by each trial's target duration, a red disk (3° diameter) was presented at the same location for 200 ms. Participants were asked to attend to the interval between both stimuli.

After a 1500 ms delay following the disappearance of the red disk, the white disk was presented again at a different random location within the same area for 200 ms, this cued the beginning of the reproduction phase. Here, participants had to predict at what moment the offset stimulus should appear following the same interval as in the learning phase. The reproduction phase ended with participant response, which varied depending on the method used for that block (discrete or bracket reproduction).

During *discrete reproduction blocks*, participants were instructed to press the SPACE key at the exact moment when the red disk would appear following the same interval as in the learning phase.

**Figure 2.**
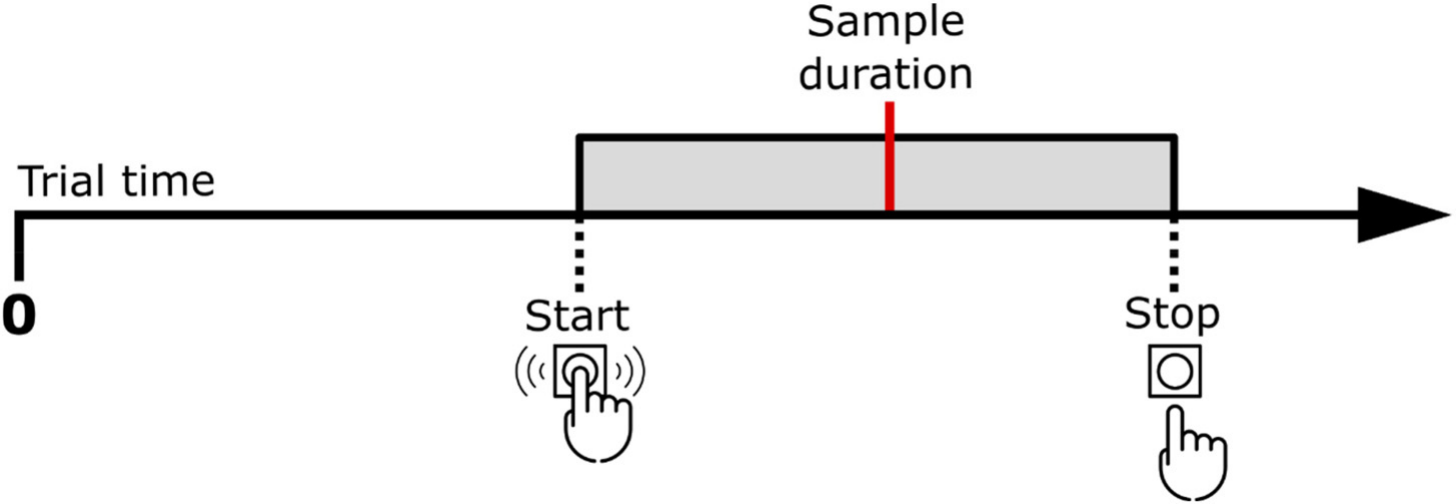
Example of the bracket encompassing the sample duration. Participants start pressing before the sample duration and release after it.

In *Bracket reproduction blocks*, the only difference was that participants were asked to press and hold the SPACE key as soon as they believed that it was probable that the red disk should appear and release it when they were confident that it would have already appeared according to the same interval as in the learning phase (see Figure 2). They were encouraged to do this continuous press as short as possible, but trying to always include the end of the target duration inside it.

We randomized the location of the learning phase and reproduction phase stimuli to prevent possible interferences due to spatial adaptation. Also, to prevent counting during this task, participants were explicitly asked not to use any counting strategy (Rattat & Droit-Volet, 2012).

For the target durations, we selected a comprehensive range of sub- and supra- second durations of 600, 900, 1300, 1900, 2700, and 4000 ms.

Each participant performed three blocks using the discrete reproduction method interleaved with three blocks using the Bracket reproduction method. The order of the blocks was counterbalanced between participants. All blocks consisted of 60 trials that included 10 repetitions of each target duration in a random order. This added up to a total of 60 repetitions of each target duration half of them using the discrete reproduction and the other half using the Bracket reproduction.

### Measures

Reproduction times were measured differently depending on the reproduction method used. In all cases, the reproduction times started after the presentation of the white disk in the reproduction phase.

In the discrete method blocks, reproductions were considered as the elapsed time until participant's first pressing of the SPACE key.

In the Bracket method blocks we obtained two points directly from participants response, the start of the bracket as the elapsed time until the first pressing of the SPACE key and the end of the bracket as the elapsed time until its release. From these two measures, we calculated the bracket length as the elapsed time between start and stop times and the reproduction time (to be compared analogous to the one obtained in with the discrete method) as the elapsed time until the midpoint between start and stop times.

The selection of the midpoint of the bracket as the reproduction time could be argued since other moments in the bracket could be closer to the target duration. The reasoning is that, following Kladopoulos et al. (1998), it is the midpoint of the bracket that should be analogous to the PSE, which represents the perceived target duration.

### Predictions

First, we will compare whether the Bracket reproduction method is a valid alternative to the discrete reproduction method by comparing its accuracy and precision at each target duration. We will also assess whether both methods exhibit robust features of time perception, such as scalar invariance in a similar manner. We expect similar performance when comparing the reproduction time obtained from the discrete reproduction method with the midpoint of the bracket from the bracket reproduction method.

Then, to understand how the Bracket is positioned, we will investigate its relation with start and stop times. Finally, to study what the Bracket length represents, we will calculate its correlation with the variability in reproduction estimates, as well as how it might change along different target durations, two sources of information that are supposed to represent uncertainty. In this regard, we expect to find a positive relation between bracket length and variability of reproduction estimates, and according to the scalar property of time, we also should see an increase in both as duration magnitude increase.

### Modeling

In timing tasks like those we mentioned above, participants engage in a continuous process of comparing the current subjective elapsed time with the representation of a target interval or the criterion time. As time progresses, the discrepancy between these two intervals gets narrowed until it reaches a threshold at which both are perceived to be equivalent. If the elapsed time continues beyond this point, the discrepancy begins to rise again, reaching a second threshold after which intervals are no longer perceived as equal (Gibbon & Church, 1990; Kladopoulos et al., 1998).

This description of the process highlights how important it is not only to keep a good track of the current elapsed time and maintain a stable representation of the target interval, but also that the relation between these thresholds and the magnitude of the stimuli is critical to understanding the perceptual decision process.

**Figure 3.**
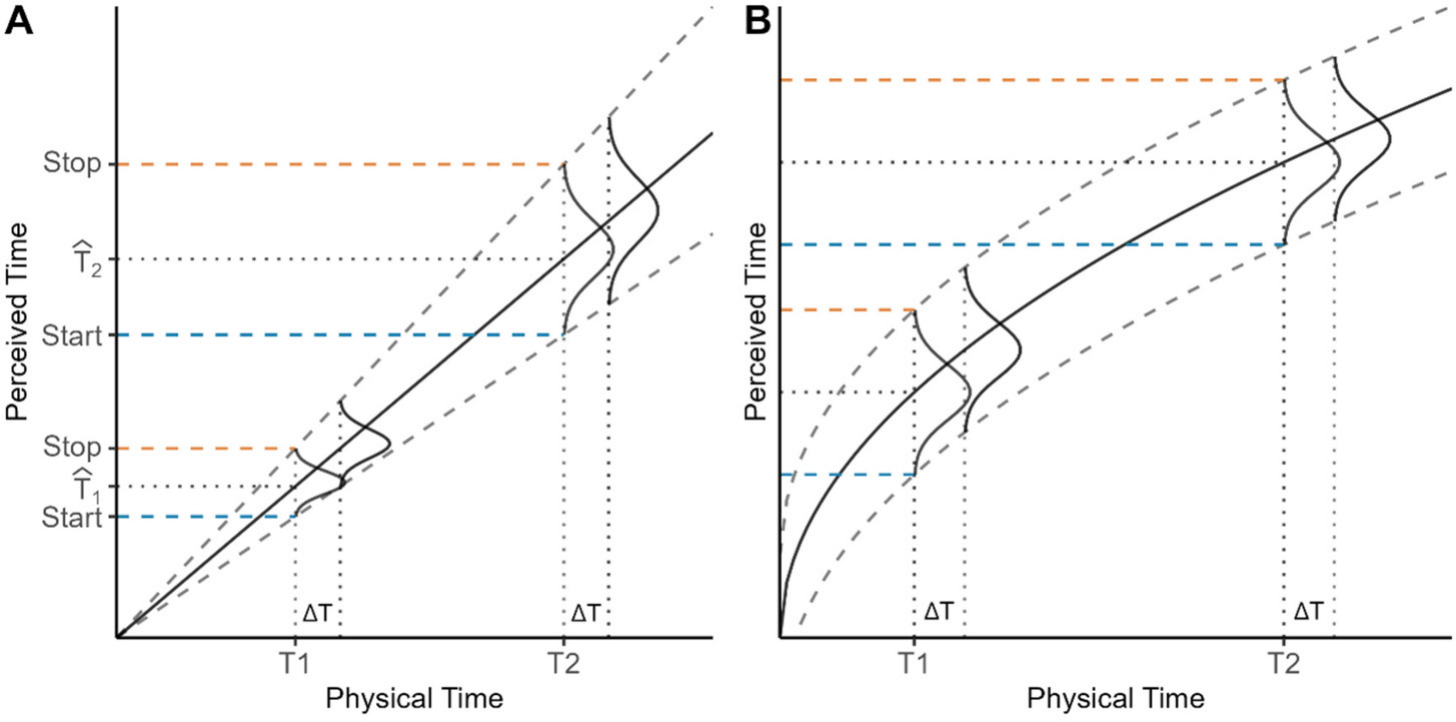
Example of the increase in threshold range (dashed lines) in relation to the transducer (solid lines). The start and stop boundaries of the bracket (blue and orange lines respectively) represent a proxy for this range. (A) Example of a multiplicative increase in noise. The same increase in physical magnitude (ΔT) at T1 and T2 is harder to discriminate at longer durations (T2) due to the increase in noise. (B) Example of an additive noise (independent of magnitude). The power function transducer makes the same increase in magnitude (ΔT) harder to discriminate at longer durations, but this time due to the compression of the transducer.

Gibbon and Church (1990) propose that the discrepancy needed between the current subjective time and the remembered time is proportional to the remembered time. This implies that thresholds diverge further from the actual target duration in relation to its length, reducing sensitivity due to a multiplicative nature of noise and aligning it with the scalar property of timing (see different durations in Figure 3A). However, if we conceive the scalar property of timing as a form of Weber's Law, there are multiple combinations of noise and transducer (understood as the representation of the relationship between real and perceived duration) that could still hold with this law (Zhou et al., 2024). For example, a transducer that follows a power function with an exponent smaller than 1 would comply with Weber's Law when additive noise is present (Zhou et al., 2024) (see Figure 3B). Therefore, the scalar property could still hold depending on what is the relationship between noise and magnitude and how the perceived time evolves as physical time increases.

In the context of our study, we conceive the bracket length as a probe into the noise, and the start and stop times could specifically reflect the moments at which each threshold is reached, reflecting the nature of the noise that determines these thresholds.

Regarding the process of reaching these thresholds and according to ramping activity or pulse-accumulating models, each pulse of the internal clock or each accumulation of evidence is subject to some degree of noise. This inherent variability in the accumulation process means that when the accumulated subjective time reaches a threshold, it might not precisely reflect the actual elapsed time (Gibbon & Church, 1990; Simen et al., 2016). In this sense, we hypothesize that stop times should exhibit greater variability than start times due to the necessary accumulation of more pulses, and, therefore, potentially more noise. However, despite this differential variability, we expect that the average start and stop times will not be significantly shifted due to this noise, as they should fall on average around the real threshold time.

By studying the variability of each of these components and how they evolve throughout different target durations, we aim to shed light on how the accumulation process is influenced by process noise and characterize it. This is, we propose an interpretation of what start and stop times could reveal about the nature of this process noise being multiplicative, additive, or a mixture of both.

**Figure 4.**
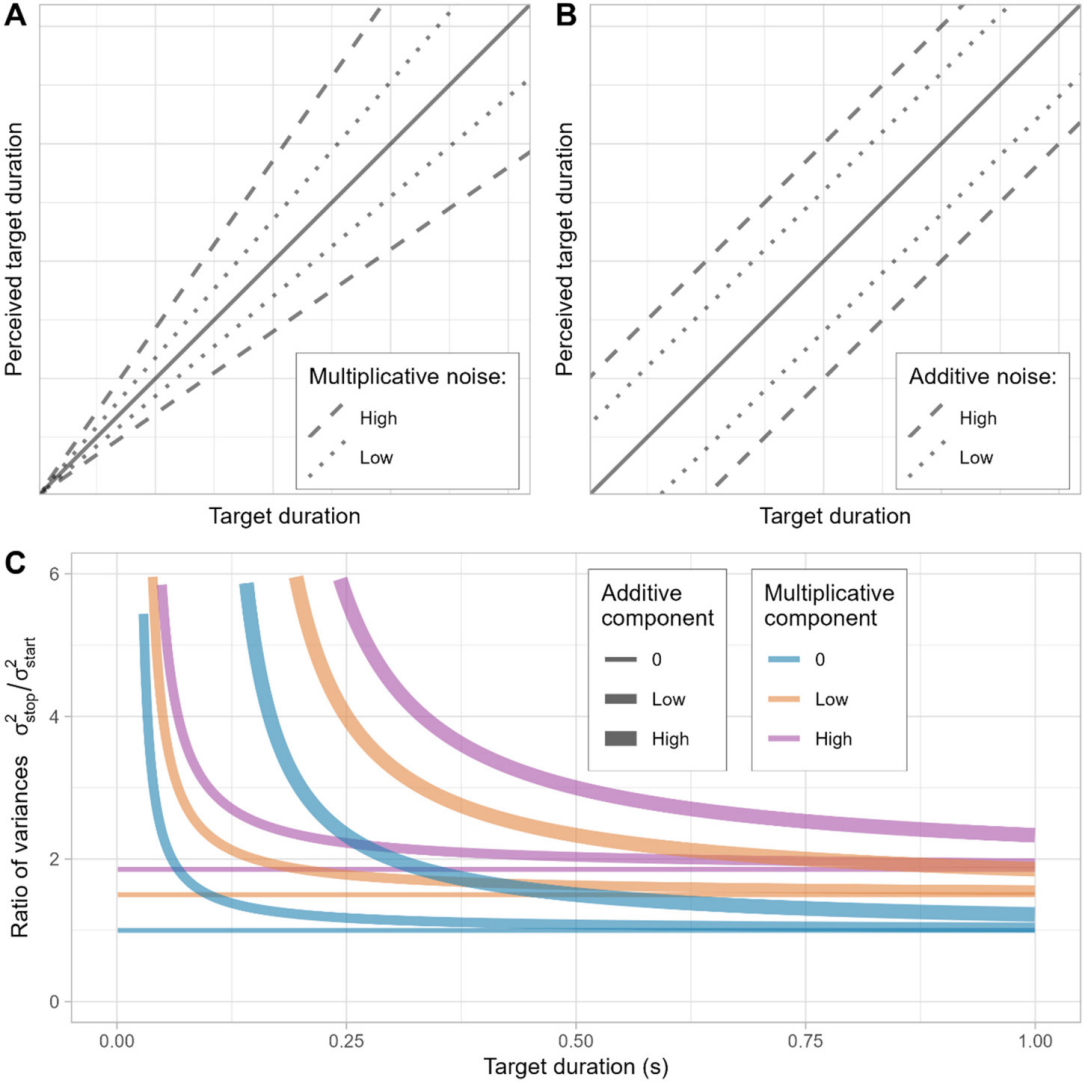
(A and B) Solid lines represent a linear transducer of how the perceived duration represents real duration. Dashed and solid lines show the spread of perceived times due to different sources of noise. (A) Multiplicative noise correlates with the magnitude of the stimuli. Increasing variability at longer durations. (B) Additive noise is independent of stimulus magnitude and is stable across the range of durations. (C) Simulations of the ratio of variances with different levels of additive and multiplicative threshold components. The rightward asymptote is mostly determined by the multiplicative threshold and the approximation to this asymptote is modulated by the additive threshold.

For this, we propose a definition of the distance from start or stop time to the center of the bracket according to two components (see Equation 1): an additive component (*c_a_*) that has a fixed value and that could be related to the minimum time participants allow themselves to leave between start and stop times and a multiplicative component (*c_m_*) that increases proportionally to the perceived duration ( $\hat{t}$ ). If thresholds are only determined by an additive component, the bracket length should be relatively constant across durations, whereas an addition of the multiplicative component would predict a proportional relationship between duration and bracket length (see Figure 4A and 4B for an example of the contribution of these components to the start and stop range with different transducers and natures of noise).

(1) $$Bracket=c_{a}+c_{m}\cdot \hat{t}$$

Additionally, based on the idea of the accumulating noise in the ramping activity, we can study how start and stop times differ in terms of variability and how it could reflect a prevalence of additive or multiplicative noise. If we assume a multiplicative noise, we should find that the ratio obtained from dividing the variance of stop times by the variance of start times would remain constant independently of the target duration. This is because the increase in variability proportional to the target duration would increase in a proportional manner in both estimates. In contrast, if there is a presence of additive noise, which is independent of the target duration, it would impact both start and stop times to the same degree independently of target duration and would lose effect as durations get longer and the relative weight of this noise becomes negligible. This would manifest by decreasing variance ratios as target durations increase.

A combination of both types of noise could also be accounted by the model, and the relationship between target duration and ratios of variances would manifest a horizontal asymptote according to the multiplicative noise (or to 1 if there is only additive noise), and the slope toward this asymptote would be determined by the additive noise component (see Figure 4C for an example of different combinations).

## Results

### Data Filtering

We filtered out those trials where reproductions or bracket lengths (in bracket method blocks) differed by more than 2 standard deviations from the average calculated for each participant, method, and duration. This was done to remove non-valid trials where the reproduction response was delivered with no relation to the perceived duration, such as situations in which participants could have mistakenly delayed their response due to mixing up the learning and reproduction phases, or trials in which impulsive responses or involuntary releases of the key created abnormally short bracket measures or reproduction times. This produced a data loss of 7.8% from the bracket method blocks and 4.6% from the discrete method blocks.

### Performance

**Figure 5.**
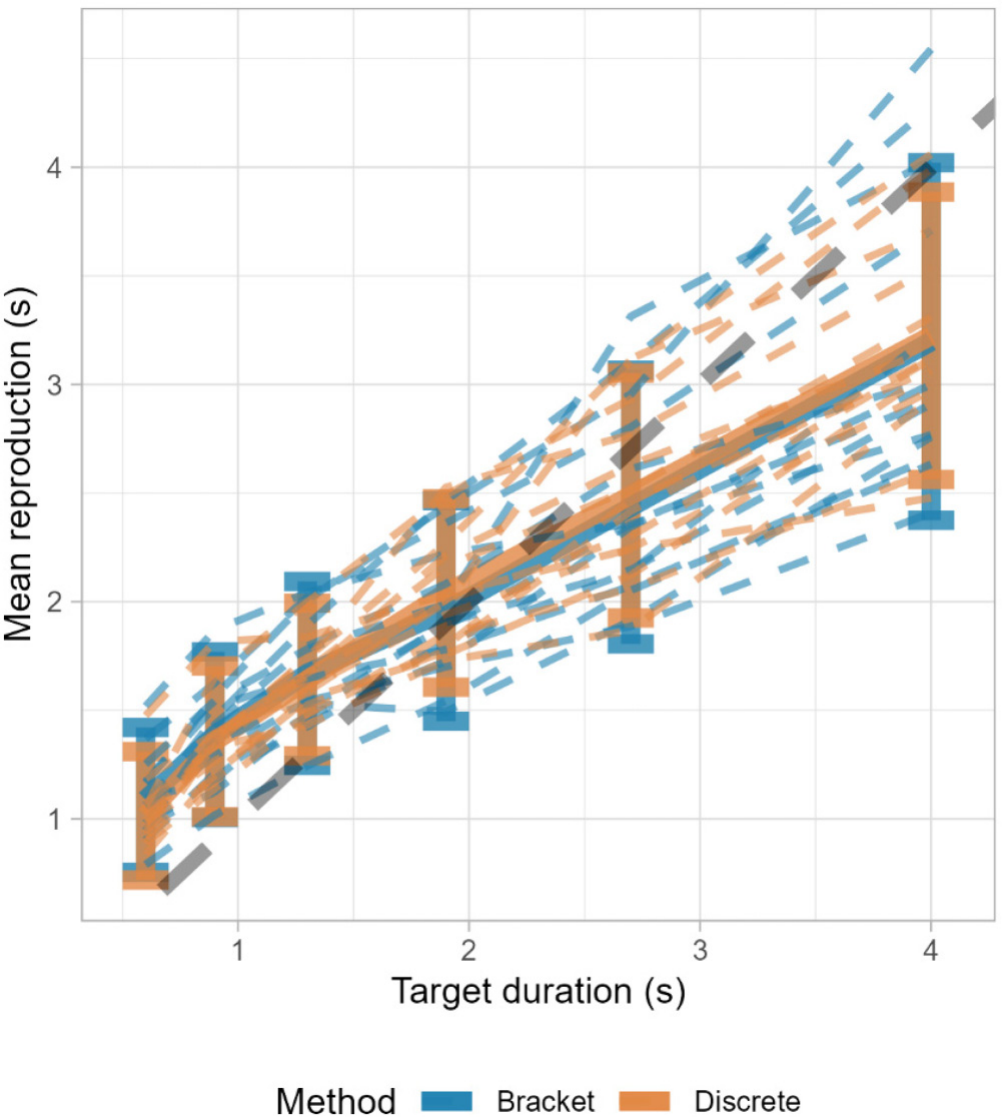
Performance between methods. Bold lines represent the aggregate average among participants. Dashed lines represent individual participants’ data.

In our initial analysis, we directly compared the time reproduction estimates obtained from the discrete and bracket reproduction methods (taking as the latter the midpoint of the bracket as the estimate). The comparison revealed a striking similarity between the two tasks in terms of the direction, magnitude, and variability of errors (see Figure 5). The overlap of the average estimates from both methods suggests that the bracket reproduction method may serve as a viable alternative to the classic approach.

Regression analysis using a power function (*y = ax^b^*) revealed a strong and significant relationship between target durations and reproduced time for both the discrete, *R*^2^* *= .74, *F*(1, 2401) = 6706, *p *< .001, with coefficients *a *= 0.31, 95% CI [0.3,0.32], *p *< .001, and *b *= 0.6, 95% CI [0.59,0.62], *p *< .001, and the Bracket, *R*^2^* *= .65, *F*(1, 2310) = 4306, *p *< .001, with coefficients *a *= 0.33, 95% CI [0.31,0.34], *p *< .001, and *b *= 0.55, 95% CI [0.54,0.57], *p *< .001, methods.

**Figure 6.**
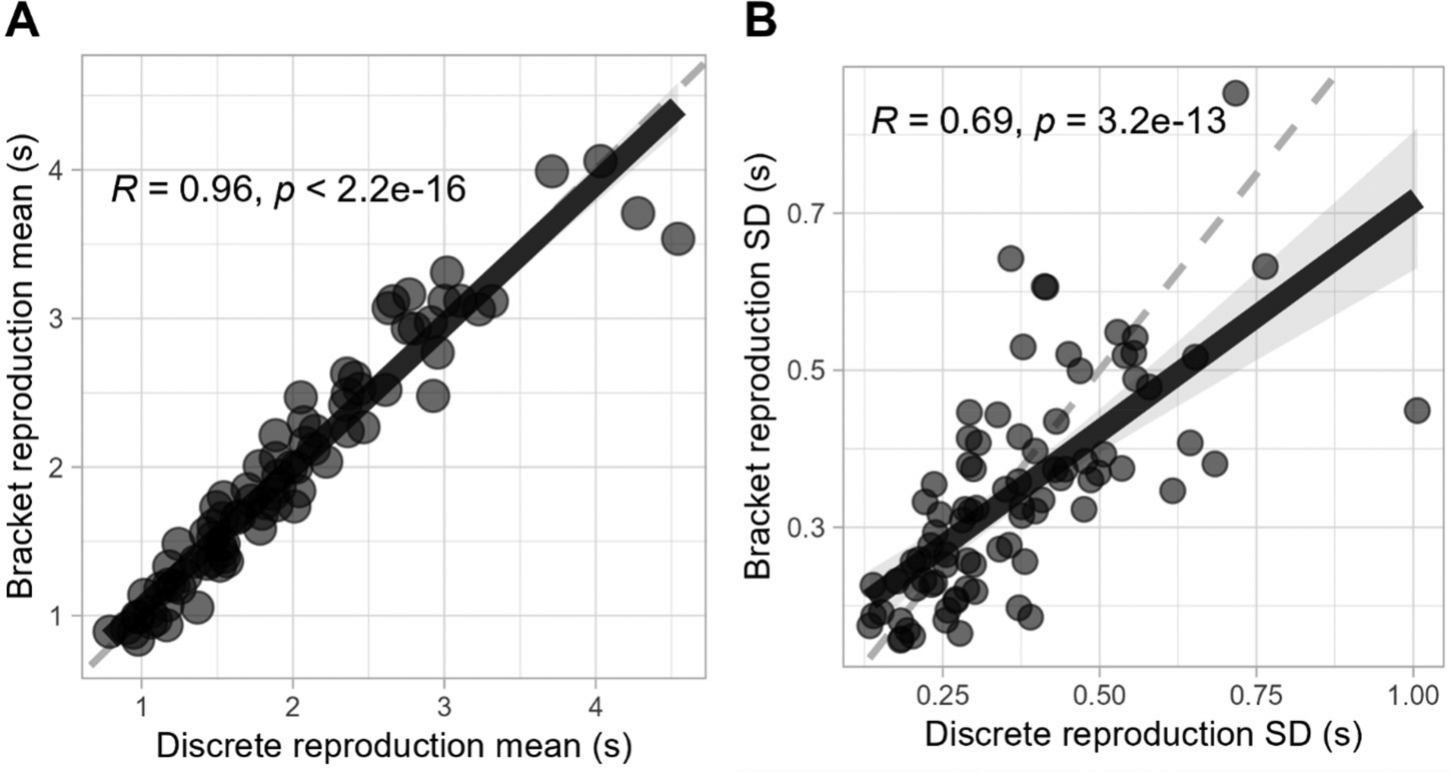
Parameter correlation between methods. Correlation of (A) reproduction means and (B) standard deviations between methods. Each data point is calculated as the average or standard deviation of reproductions for each participant and target duration.

The overlapping of reproduction estimates from both methods demonstrates a high degree of consistency between them. This was examined in more detail by measuring the correlation between the parameters of reproduction times across methods. As shown in Figure 6A, we found a remarkably high correlation for the mean reproduction times of each participant and target duration between methods, *r*(84) = .96, *p* < .001, indicating that on average, individuals reproduced time intervals with similar accuracy across both methods. Furthermore, we also found a substantial correlation for the variability of these reproductions, as measured by the standard deviation, *r*(84) = .69, *p* < .001, suggesting that the degree of precision with which individuals estimate time is also consistent across methods (see Figure 6B).

Still, correlation alone is not sufficient to confirm that the midpoint of the bracket is the optimal representation of the discrete reproduction time, as any other systematic point of the bracket could also exhibit a similar high correlation. To address this, we conducted a linear regression analysis using the average reproduction time from the discrete method as a predictor of the bracket method's average midpoint. The analysis yielded an intercept of 0.05326 (*p* = .406), indicating no significant systematic bias from zero, and a slope of 0.97138 (*p* < .001), suggesting a nearly one-to-one relationship between the discrete and bracket methods’ reproduction times. These results provide strong evidence that the bracket’s midpoint can indeed be considered a direct analog to the discrete method's reproduction time.

Our comparison between methods also considered the implications of well-known phenomena in time perception research, such as Vierordt's law and the scalar property of timing or Weber's law.

**Figure 7.**
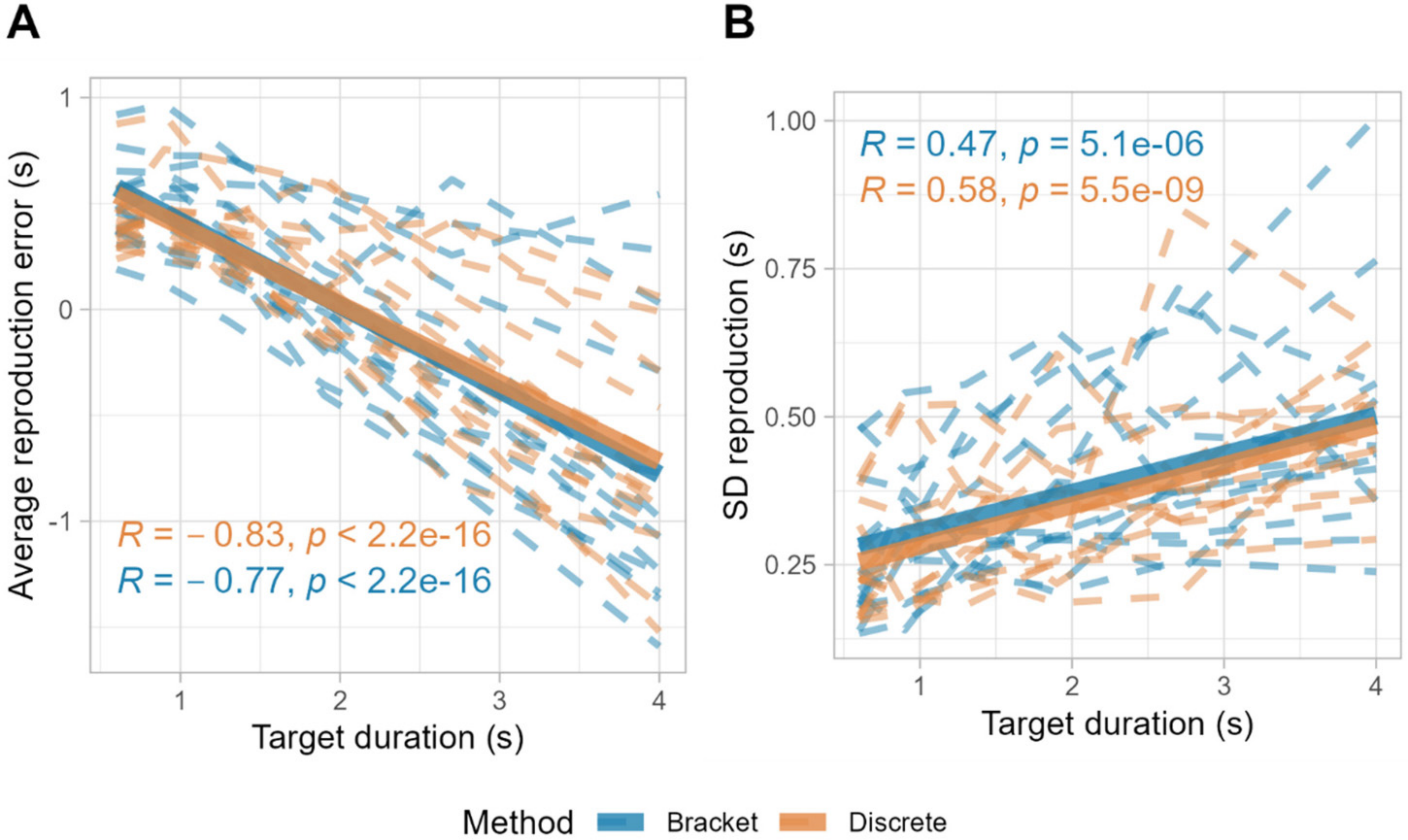
Bold lines represent the aggregate average among participants. Dashed lines represent individual participants’ data.

Consistent with Vierordt's law, we observed that in both methods shorter durations were typically overestimated while longer durations were underestimated, evidenced by a strong negative correlation between target duration and average error, *r*(84) = −.83, *p* < .001 for the discrete method and *r*(84) = −.77, *p* < .001 for the bracket method; see Figure 7A.

Furthermore, the scalar property of timing was also manifested in our data, with an increase in the standard deviation of reproductions as a function of target duration, *r*(84) = .58, *p* < .001 for the discrete method and *r*(84) = .47, *p* < .001 for the bracket method; see Figure 7B. This positive correlation aligns with the scalar timing theory, which posits that the precision of time perception is proportional to the timed interval itself (Gibbon, 1977; Malapani & Fairhurst, 2002).

This mutual adherence to Vierordt's law and the scalar property reinforces the behavioral equivalence of the two methods, suggesting that each method is equally capable of capturing these inherent phenomena if temporal estimates.

### Bracket Length as an Uncertainty Measure

Next, we wanted to assess the potential of the bracket method as a tool for measuring uncertainty in time perception. To this aim, we studied how the bracket length varies under conditions where uncertainty is expected.

First, in line with the scalar property of timing, also interpreted as the Weber's law, we hypothesized that if uncertainty is expected to increase as the magnitude of the target duration increases, the bracket length should reflect this growth by also increasing with target duration. To test this, we examined the correlation between the average bracket lengths and target durations after standardizing the bracket lengths to account for individual differences in participants’ bracketing strategies. This standardization normalizes the data within each participant, removing individual bias toward conservative or liberal bracketing and allowing us to focus on relative changes in bracket length in response to varying target durations.

By fitting a power function in a regression analysis, we found a robust positive relationship between standardized bracket length and target duration *R*^2^_adj_* *= .86, *F*(1, 82) = 518.9, *p *< .001, with coefficients *a *= 0.74, 95% CI [0.7, 0.78], *p *< .001, and *b *= 0.52, 95% CI [0.47,0.56], *p *< .001.

**Figure 8.**
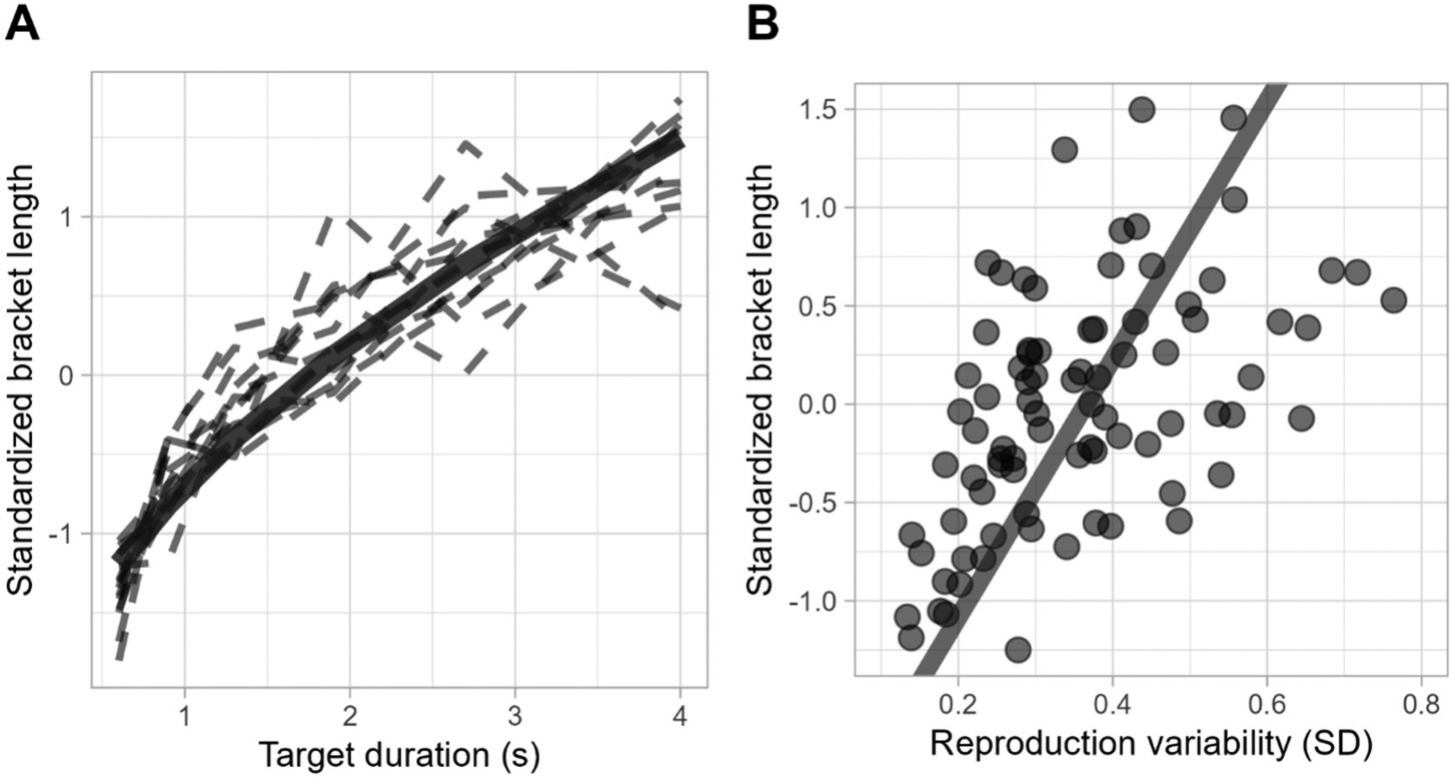
(A) Relationship between standardized bracket length and target duration. The bold line represents the general trend among participants, while the dashed lines represent individual participants’ data. (B) Deming regression of standardized bracket length as a predictor of reproduction variability.

Participants show a tendency to extend their brackets with increasing target durations (see Figure 8A), which suggests that the bracket length may indeed capture a key aspect of uncertainty, consistent with the scalar nature of time estimation.

To further verify the bracket length as a measure of uncertainty, we examined its relationship with another established indicator: the variability of time reproductions. To adjust for the influence of target duration, we normalized the bracket lengths for each participant and target duration. This standardization removes the explained variability due to the scalar property of timing. A Deming regression was employed to account for measurement errors present in both variables. The analysis revealed a significant relationship with a slope of 6.57 (95% CI [3.77, 9.36]), an intercept of −2.34 (95% CI [−3.32, −1.47]), and an error variance ratio of 0.13. These results suggest a positive relation between the standardized bracket length and the standard deviation of time estimates (see Figure 8B), indicating that longer brackets correspond to greater variability in reproductions. This pattern reinforces the bracket length's role as a reliable indicator of uncertainty.

### Variance of Start and Stop Times

To analyze the variability of start and stop times of the bracket, we fitted a generalized linear mixed model with a Gamma distribution to the ratio of variances of stop times by start times (σ^2^_stop_/ σ^2^_start_) with target duration as a fixed effect and participant as a random effect. An offset of 1 was included to directly test the hypothesis that the ratio of variances differs from 1.

**Figure 9.**
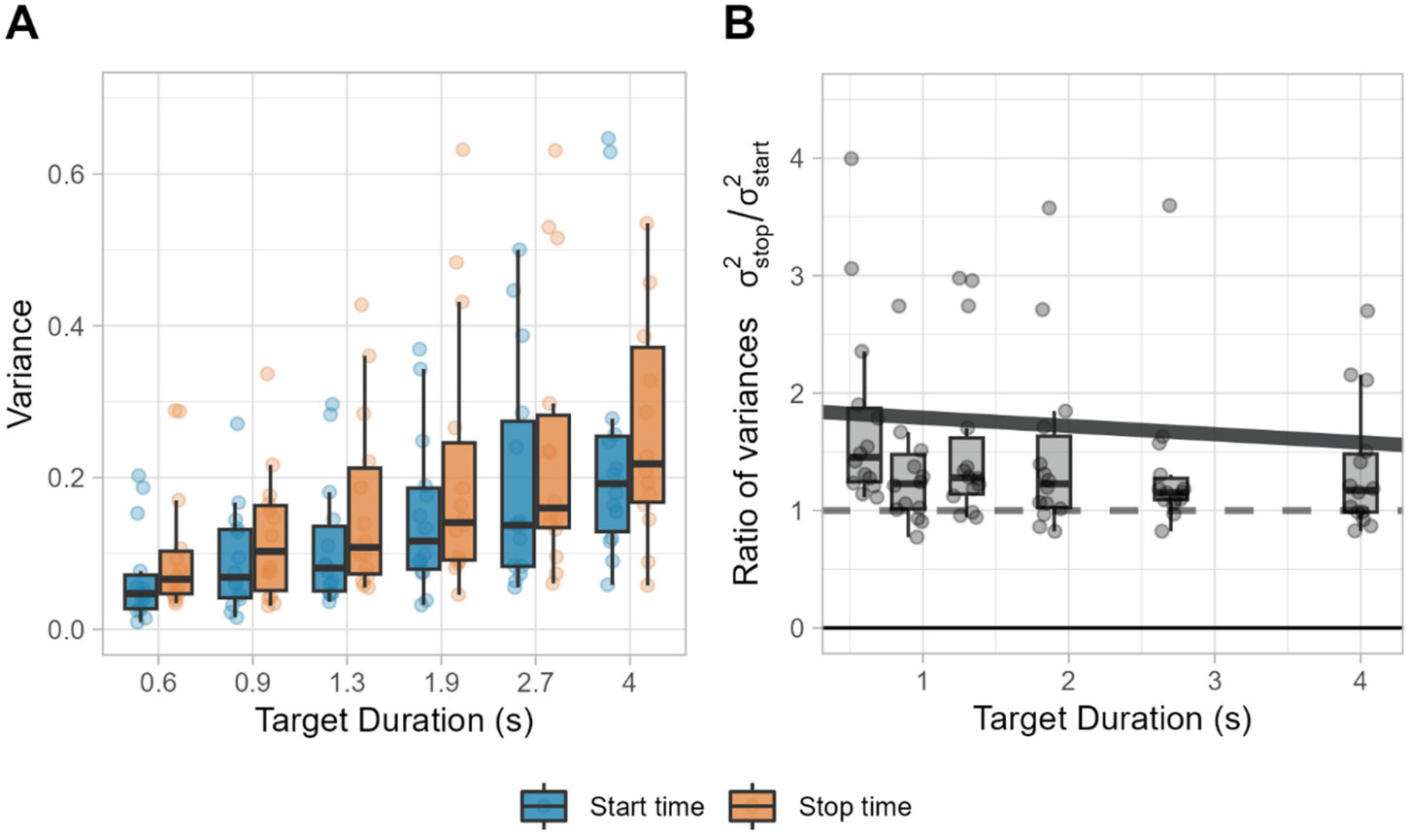
(A) Variance of start and stop times calculated from each participant and target duration. (B) Ratio of variances. Dashed line represents equal variance, and the solid line represents the predicted fit from the model.

The results showed that the intercept was significantly greater than 1 ( ${\hat{\beta }}_{0}$ = 1.86, SE = 0.27, *z *= 3.22, *p* = .001), suggesting that variance of stop time was, on average, 0.86 times greater than the variance of start times when not considering the duration effects. Moreover, the significant negative coefficient of duration (slope ${\hat{\beta }}_{1}$ = −0.07, SE = 0.03, *z *= −2.62, *p* = .009) suggests a convergence of variance ratios toward 1 with increasing target durations (see Figure 9B). This convergence suggests the presence of some additive noise in the establishment of thresholds. As mentioned before in the modeling section of the methods, an increase in the distance between thresholds (represented by start and stop times) and the center of the bracket would increase the difference in variability from start and stop times. This occurs because the threshold associated with start times is reached sooner, thus preventing the accumulation of as much noise. In contrast, the threshold associated with stop times is reached later, which means that more noise could be potentially accumulated by that time. This creates a difference between the variances of start and stop times that is especially relevant in shorter durations, but as this increase in the thresholds does not continue to grow with increasing durations, the noise already implied by these longer durations causes the absolute increase in the thresholds to be mitigated until it becomes negligible.

**Figure 10.**
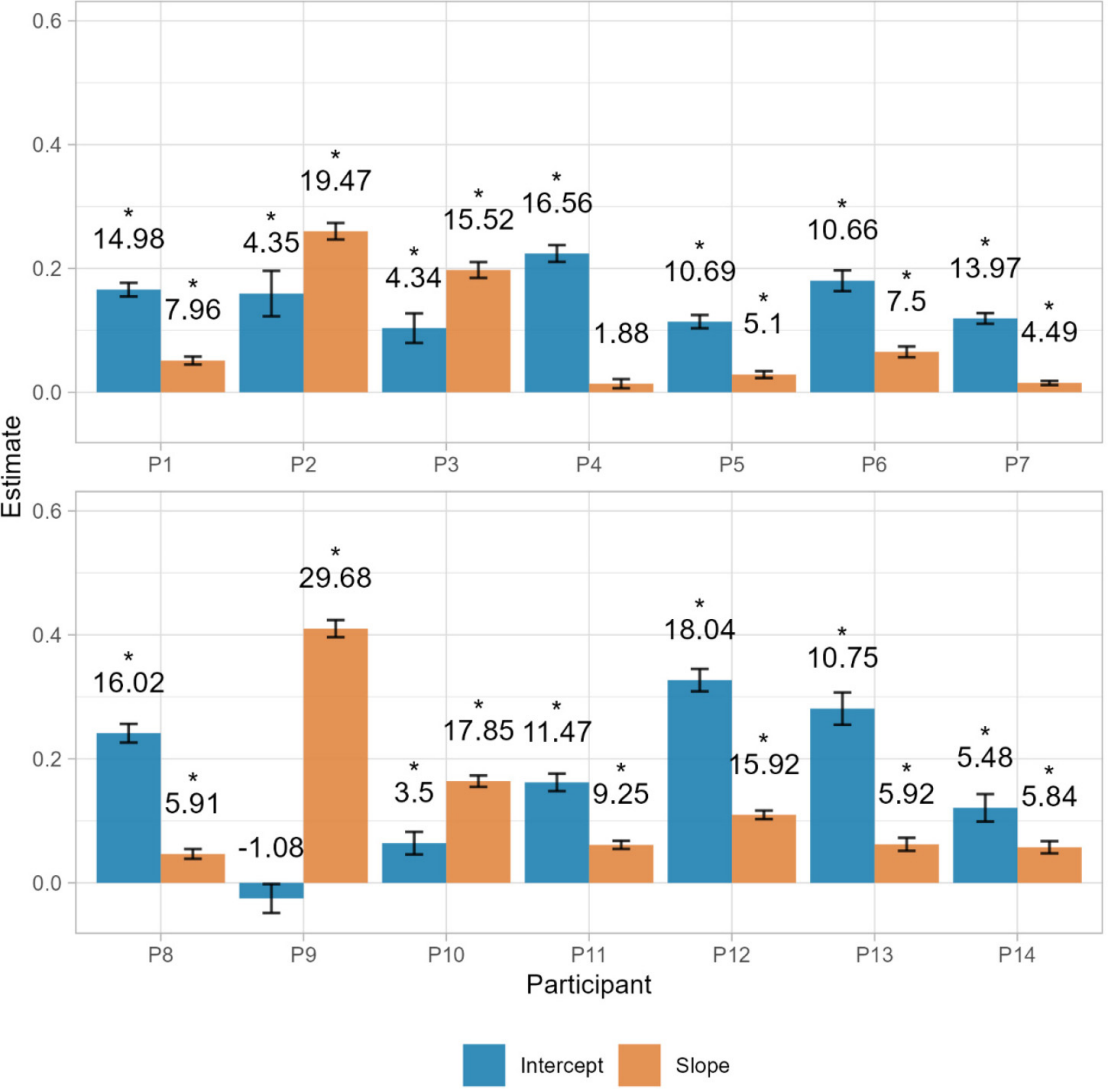
Parameters of the threshold components estimated for each participant. Intercepts represent the additive component and slopes represent the multiplicative component. Numbers above the bars represent the *t* value. Significant coefficients are marked with “*”.

**Figure 11.**
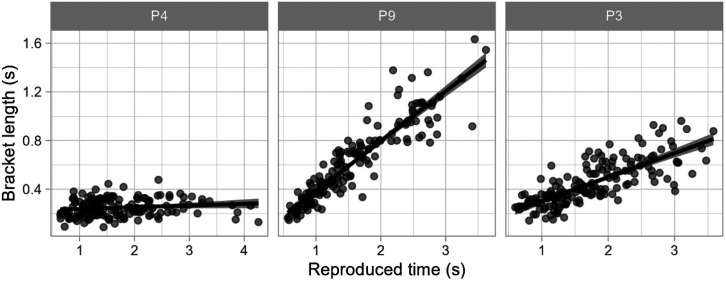
Representative examples of participants with low multiplicative, but considerable additive bracket components (P4), high multiplicative, but null additive bracket components (P9), and combination of both types of bracket components (P3).

Furthermore, following Eq. 1, we assessed the individual contribution of additive and multiplicative components of the bracket by fitting a linear model of the reproduced duration as a predictor of the bracket length. Following the rationale of the model, the intercept parameter of the model reflects the amount of bracket that is constant, regardless of duration and the slope parameter reveals the proportion of the target duration that is also added to these boundaries. We find that for almost all participants both the slope and the intercept were significantly different than 0 (*p *< .001) (see Figure 10). By looking at the parameter values, we can see that depending on the participant the contribution of additive or multiplicative components can differ, with participants with a high baseline bracket, but very little increase with longer durations and some others that have a stronger effect of duration on the spread of the bracket (see Figure 11 for some examples of these profiles).

## Discussion

In our study, we aimed to assess the capabilities of the Bracket method of reproduction and compared it directly with the conventional discrete reproduction method. First, we wanted to evaluate whether both methods are equivalent in terms of precision and accuracy, as well as their adherence to common timing phenomena. Unlike the discrete method, which provides a single point estimate, the Bracket method offers two time points. These points not only enrich our data, but also generate a bracketed interval that offering a fresh perspective on how we measure participants’ uncertainty in time estimations.

Our second objective focused on investigating the bracket interval as a potential measure of uncertainty. Traditional methods have their challenges in capturing the subtle nuances of uncertainty in time estimation. By adopting a more direct approach with the bracket method, we aimed to bypass some of these limitations and provide alternative ways of measuring time perception.

### Equivalence of Methods

Our results confirmed the equivalence between the discrete and bracket reproduction methods.

Initially, one might argue that requesting a range instead of a discrete response could alter the participants’ reproduction process. Contrary to these concerns, our findings demonstrate that participants’ performances were remarkably consistent across both methods. This consistency was evidenced by the high correlation between reproduction times from discrete responses in the discrete method and the midpoints of brackets. Similarly, the variability of these estimates remained consistent across methods, underscoring that the apparent complexity of the response did not detract from precision.

Additionally, the estimates obtained from both methods conformed to the traditional conception of the scalar property of timing, where the variability of discrete reproductions and midpoints of the bracket scaled linearly with the target duration (Gibbon & Church, 1990; Rakitin et al., 1998). Equally important, the presence of Vierordt's law was consistent across methods, with shorter durations typically overestimated and longer durations underestimated. This phenomenon reveals a central tendency effect, whereby the discrete reproductions in the discrete blocks and the shift of the bracket in the bracket blocks exhibited the same bias. These parallel outcomes not only support the interchangeability of both methods but also affirm that the adaptation of requesting participants to bracket target time does not compromise the assessment of participants’ time perception and its associated phenomena.

### Bracket Length as a Measure of Uncertainty

Building on the equivalence established between discrete and bracket reproduction methods, our study aimed at exploring the bracket methods’ contribution, specifically its potential as a direct measure of uncertainty in time perception. Traditionally, uncertainty in reproduction tasks was inferred from the variability across multiple reproductions. Our findings, however, suggest that the bracket length obtained in a single trial can already represent the variability of multiple discrete reproductions of that same target duration, offering a more efficient measure of uncertainty in reproduction tasks. Other methods tried to reach a measure on a single trial basis, such as the PI procedure (Rakitin et al., 1998), but it required many responses throughout one single trial and often the application of reward dynamics too, which could not be applicable in some studies.

Another way of relating the bracket length to uncertainty is by considering one the most unanimous principles in timing research, the scalar property of timing. It posits that uncertainty increases proportionally with the magnitude of the timed stimuli (Gibbon & Church, 1990; Rakitin et al., 1998). Our results not only align with this principle, as both discrete reproductions and the midpoints of the bracket are more variable as durations increase, but also showed that the bracket length increased along with the target duration. Consequently, participants produced longer brackets in those conditions where more uncertainty is expected. This suggests that based on the scalar property of timing the bracket length could be a potential measure of uncertainty.

These findings are the key to understand what the bracket length represents. In this regard, we also explored how the bracket could be defined under the basis of ramping activity or pulse-accumulating models, which suggest that perceptual decisions are based on the accumulation of subjective time to certain thresholds. We proposed that the bracket components could directly reflect these decision thresholds, being the starting point of the bracket the moment when the first threshold is reached and the end point of the bracket the moment at which differences are enough to be discriminated. The variability observed between the start and stop times showed that stop times were significantly more variable than start times. This difference could be plainly explained as the effect of noise in the accumulation process until reaching the thresholds since stop times necessarily require more accumulation, and it allows for more introduction of noise (Simen et al., 2016).

However, the relative difference between these variabilities decreased as target durations increased (Figure 9B). This opens an interesting avenue for discussing the nature of process noise. If the noise in the accumulating process is to some degree constant, the absolute difference in variability would increase linearly at both start and stop times, but the relative difference between them would decrease proportional to the duration's magnitude. Our findings of a decrease in the relative difference between the variabilities of start and stop times as durations increase aligns with the presence of additive noise. This was supported by the results of the bracket model by a significant presence of both additive and multiplicative components in almost all participants.

Such findings not only corroborate the hypothesis that both additive and multiplicative noises could be integral to the timing process, but also show that an interaction between these types of noise could be possible.

Moreover, it highlights the potential of the bracket method to also dissect the contributions of additive and multiplicative noise in timing estimation and even help characterize the transducer function for duration in a unique and novel way.

We exposed how the bracket method can be linked to other ways of assessing uncertainty with quantitative tasks, but decisional tasks can also be used to assess uncertainty in time perception. In these kinds of tasks that often require comparison or discrimination of time intervals, a psychometric function is typically derived, providing a measure of sensitivity from its slope (Wichmann & Hill, 2001). Since this measure of sensitivity is directly related to uncertainty, it would be interesting to assess its link with the bracket length. A relation between these two measures could provide for an advantageous alternative in scenarios that require sensitivity measures but that would benefit from a quantitative approach. This possibility opens new avenues for studying the potential of the bracket method.

Another approach that could be related to uncertainty in time perception involves confidence judgments. Confidence judgments, often employed in psychophysics tasks to assess the metacognitive level of task performance, provide a direct window into participants’ perceived reliability on their own estimations. For example, Lamotte et al. (2017) found that confidence judgments correlate with the accuracy of duration estimates in a temporal generalization task. Akdoğan and Balcı (2017) showed that individuals could introspectively assess their timing errors, linking this awareness to confidence levels. Cropper et al. (2024) and Corcoran et al. (2018) explored second-order confidence judgments in modified temporal-bisection tasks where participants had to compare their confidence levels on different estimations, allowing the assessment of time perception and confidence across multiple estimations at the same time. Jovanovic et al. (2023) further revealed that dopamine depletion affects both timing accuracy and confidence, underscoring the neurochemical underpinnings of these processes.

Although the focus of our study is not to directly discuss the relationship between confidence and uncertainty, the emerging interest in metacognitive assessments demonstrated by these studies suggests a valuable direction for future research. Specifically, exploring how the bracket method's measure of uncertainty correlates with participants’ confidence judgments could offer deeper insights about uncertainty in time perception at different stages of cognition and shed light on how much uncertainty are we actually aware of.

Furthermore, considering the bracket method in conjunction with confidence judgments and discriminability from psychometric functions presents a comprehensive approach to studying time perception. This multifaceted methodology could illuminate the complex dynamics between objective uncertainty, decisional processes, and metacognitive judgments, enriching our understanding of how individuals perceive, estimate, and reflect on their own experience of time.

### Conclusion

The present study has demonstrated that the bracket reproduction method is not only a viable alternative to the classic discrete reproduction method, but also a superior tool for directly measuring uncertainty in time perception. We have shown that both methods can yield equivalent results in terms of accuracy and precision at reproducing intervals from 0.6 up to 4 s. Moreover, our findings suggest that the bracket length could serve as a powerful indicator of uncertainty, opening new avenues for research in time perception. Also, the study of the components of the bracket provides a unique way to address the discussion about additive or multiplicative noise in perceptual estimations. With this, the bracket method presents itself as a significant improvement of the methodological toolkit in time research that can allow us to better understand the variability inherent in human time perception and provide insights into the cognitive processes underlying timing behavior.
